# The Reversal Effect of Sigma-1 Receptor (S1R) Agonist, SA4503, on Atrial Fibrillation After Depression and Its Underlying Mechanism

**DOI:** 10.3389/fphys.2019.01346

**Published:** 2019-11-14

**Authors:** Xin Liu, Chuan Qu, Shaobo Shi, Tianxin Ye, Linglin Wang, Steven Liu, Cui Zhang, Jinjun Liang, Dan Hu, Bo Yang

**Affiliations:** ^1^Department of Cardiology, Renmin Hospital of Wuhan University, Wuhan, China; ^2^Cardiovascular Research Institute, Wuhan University, Wuhan, China; ^3^Hubei Key Laboratory of Cardiology, Wuhan, China

**Keywords:** sigma-1 receptor, depression, conduction, inflammation, atrial arrhythmia

## Abstract

**Aim:**

Sigma-1 receptors have been investigated and shown to play a protective role in both depression and cardiovascular disease. SA4503, known as a σ1 receptor agonist, regulates cardiac calcium and potassium channels in rat models of depression. However, it remains unknown whether SA4503 can alleviate myocardial inflammation or conduction junctions in the atrium after exposure to chronic mild stress.

**Methods and Results:**

Sprague-Dawley male rats received 28-day treatment with SA4503, simultaneously with chronic mild stress. Behavior measurements were assessed after the daily doses. Additionally, a multielectrode array assessment, electrophysiological study, immunohistochemistry analysis, histological analysis, and Western blot analysis were performed. Depression rats’ hearts showed abnormal electrical activity, including disordered excitation propagation and prolonged total activation time (TAT). In addition, atrial arrhythmias (AAs), induced by burst stimulation, showed higher incidence and longer duration in the depression group compared to the control group. These changes were related to reduced conduction junctions and enhanced spatial heterogeneity. Importantly, depressed rat hearts showed greater expression of inflammatory factors (TGF-α, IL-6, and TGF-β), more collagen distribution in the extracellular matrix, and lower expression of gap junction proteins (CX40 and CX43). Furthermore, SA4503 partially mitigated the above indices in the depression group (*P* < 0.01 for all groups).

**Conclusion:**

These findings show the effects of the σ1R agonist SA4503; it alleviates atrial myocardial inflammation and conduction junctions after chronic mild stress. SA4503 may be the promising pharmacological agent to treat depression-related AAs by increasing conduction function, improving the expression of connexin 40 and 43, and reducing cardiac myocardial inflammation.

## Introduction

The sigma-1 receptor (σ receptor, S1R) is a molecular chaperon protein that is widely expressed in numerous tissues, including the brain and heart, and localizes on the endoplasmic reticulum (ER), which borders the mitochondria (Hayashi and Su, 2007). SA4503, recognized as an S1R agonist, has been reported to exhibit antiarrhythmic as well as antidepressant effects in a depression model (Maurice and Su, 2009). Our previous study found that S1R activation produced antiarrhythmic effects with reversed expression of voltage-dependent L-type calcium channels and potassium channels in a rat model of depression. In addition, S1R stimulation reduced sympathetic activity, which may be potential mechanistic contributors (Liu X. et al., 2018).

Atrial arrhythmias is a world-wide cardiovascular epidemic, affecting more than 33 million people across the globe (Piccini and Daubert, 2014), among which AF accounts for a significant proportion. Numerous studies report that AF is highly associated with incident depression. Depression, as an independent risk factor, provides prerequisites for the formation and maintenance of AAs (Frasure-Smith et al., 2009; Shi et al., 2017a; Walters et al., 2018). Atrial electrophysiological abnormalities, structural abnormalities, autonomic remodeling, and inflammation are the prevailing mechanisms leading to AAs (Wakili et al., 2011). Recent studies have established that inflammation might create an environment for the progress of AF. Inflammatory factors such as TNF-α participated in the initiation and perpetuation of AF while AF could accelerate immune responses, causing an “AF begets AF” phenomenon. TNF-α, secreted mainly by activated macrophages, plays a vital role in atrial fibrosis and redistributes the expression of connexin 40 (Cx40) and connexin 43 (Cx43), contributing to local conduction disturbances (Sawaya et al., 2007). Despite existing findings regarding AAs after depression, it remains unknown whether SA4503 can alleviate myocardial inflammation or conduction junctions in the atrium after exposure to depression.

## Materials and Methods

### Animals

A total of 56 adult male Sprague-Dawley rats were randomly divided into four groups, each weighing between 200 and 250 g. The first group received intragastric administration of 0.9% saline as a control (CTL; *n* = 14), the second received 0.9% saline + SA4503 (CTL + SA4503 group, CTS; 0.3 mg/kg/d, MCE; *n* = 14), the third received 0.9% saline during 4-week CUMS (the major depressive disorder group; MDD; *n* = 14), and the fourth received CUMS along with SA4503 (the MDD + stimulation group, MDS; 0.3 mg/kg/d; *n* = 14). SA4503 is a selectively non-competitive S1R agonist that confers protective effects. All experimental procedures were consistent with the Guide for the Care and Use of Laboratory Animals as published by the United States National Institutes of Health and were approved by the animal experimental administration of Wuhan University, China (No. 20160103).

### Depression Model Procedure

**FIGURE 1:**
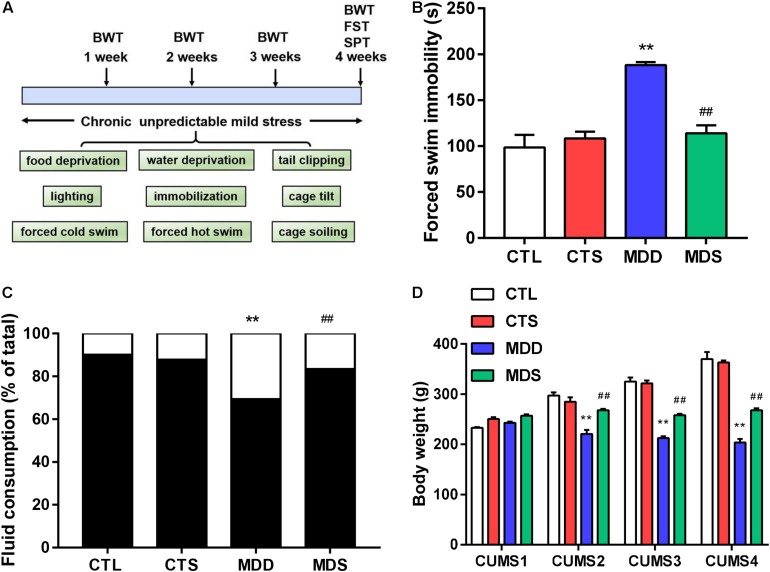
Timeline and behavioral measurement results. **(A)** Timeline of procedures and stressors used in this study; **(B)** immobility time (higher in the MDD group after the procedure); **(C)** sucrose intake after 28 days of CUMS (significantly lower in the MDD group compared to the CTL group, but partially recovered in the MDS group); **(D)** body weight loss in the MDD group induced by CUMS in weeks 1, 2, 3, and 4; ^∗∗^*P* < 0.01 vs. CTL group; ^##^*P* < 0.01 vs. MDD group. CUMS, chronic unpredictable mild stress; SPT, sucrose preference test, FST, forced swimming test, BWT, body weight test.

Chronic unpredictable mild stress was used to induce depression in rats, according to previously described methods (Liu et al., 2017). For 28 consecutive days, rats were randomly contacted with one of the following stressors: 24-hour food deprivation, 24-hour water deprivation, 10-minute hot swim at 40°C, 10-minute cold swim at 4°C, 1-minute tail clipping, 24-hour damp bedding, 24-hour lighting, 24-hour darkness, 24-hour 45°cage tilt, or 1-hour restricted mobilization, to maximize the unpredictability of the stressors (Figure 1A).

### Behavioral Measurements

Behavioral measurements consisted of three tests: the sucrose preference test (SPT), forced swimming test (FST), and the body weight test (BWT) (Liu X. et al., 2018).

#### Sucrose Preference Test

This test was used to operationally define anhedonia. For the first 20 h, rats were provided neither food nor water. Two pre-weighted glass bottles, one containing 1% sucrose and the other containing pure water with same weight, were placed in the cages. An hour later, the bottles were weighed again and the results were calculated as follows:


$$\mathrm{Sucrose}\mathrm{preference}\left(\%\right)=\frac{\mathrm{Sucrose}\,\mathrm{consumption}}{\mathrm{Sucrose}\,\mathrm{consumption}+\mathrm{Water}\,\mathrm{consumption}}$$

#### Body Weight Test

This parameter represented a diagnostic criterion for depressive-like behavior. The body weight of each rat was measured and recorded by electronic balance at the same time at the end of every week, and the effects of CUMS on body weight were analyzed.

#### Forced Swimming Test

This represented another core diagnostic criterion indicating learned helplessness and reactions to severe conditions. Each rat was placed in an unescapable transparent circular tube with a height of 50 cm and a diameter of 20 cm. Water was filled into the cylinder with a depth of 40 ± 1.5 cm at room temperature. Rats were pre-positioned to swim adaptively for 2 min and filmed without awareness of being monitored for the whole 6 min. Immobility time was defined as stillness of entire body apart from small movements for balance during the last 4 min. Each rat was removed, dried, and returned to the cage at intervals, and the cylinder was washed between tests to eliminate the smell of rat.

### Electrophysiological Study

#### Isolated Heart Preparation

Electrophysiological changes in the atrium were measured in four groups of rats. The rats (*n* = 6 in each group) were anesthetized with intraperitoneal pentobarbital sodium (40 mg/kg, Sigma-Aldrich, St. Louis, MO, United States) and administered heparin sodium (400 U, Sigma-Aldrich, St. Louis, MO, United States). The hearts were removed and immediately cannulated into the Langendorff perfusion system (ADInstruments, Dunedin, New Zealand) with oxygenated buffer solution (135 mM NaCl, 5.4 mM KCl, 1.8 mM CaCl_2_, 1.0 mM MgCl_2_, 0.3 mM Na_2_HPO4, 10 mM HEPES, and 10 mM glucose adjusted to pH 7.4 with NaOH) for 20 min. The constant pressure was maintained at 70–90 cm H_2_O and temperature at 37.0 ± 0.5°C. Irreversible myocardial ischemia was defined as ST segment elevation that continued for at least 30 min and/or part of the myocardial regions exhibiting white discoloration.

#### MAP Analysis

The experimental stimulation protocol was performed as previously described (Shi et al., 2014). Monophasic action potentials were measured from the LAA. Two custom-made Ag-AgCl electrodes with 0.25 mm diameter and 0.5 mm spacing were matched with platinum stimulating electrodes with 0.25 mm diameter and 1 mm spacing. The stimulating electrodes discharged 2-ms square-wave pulses with amplitudes twice the diastolic threshold. The distance between the Ag-AgCl electrodes and the platinum electrodes was strictly fixed to 1 cm in all groups. S1S1 overdrive protocols were used to detect the BVR, reflecting repolarization from heart rate variation. Rapid S1S1 pacing indicated that PCL gradually decreased from 150 ms to 60 ms at a rate of 10 ms lasting for 30 s to ensure stability, after which it stopped for at least 30 s to minimize the pacing memory effects. The APD at LAA were determined at 90% repolarization (APD90). This lasted for 31 consecutive times to steady APD90 before AF or electrical alternans were used to calculate BVR, the STV, and LTV. The STV and LTV were calculated as follows:


$$\mathrm{STV}=\frac{\sum \left|\mathrm{APD90}\,\left(n+1\right)-\mathrm{APD90}\,\left(n\right)\right|}{30\times \sqrt{2}}$$

$$\mathrm{LTV}=\frac{\sum \left|\mathrm{APD90}\,\left(n+1\right)+\mathrm{APD90}\,\left(n\right)-2\,\mathrm{APD90mean}\right|}{30\times \sqrt{2}}$$

The PES protocol was used for the atrium effective refractory period, which was composed of eight stimuli (S1) stimulation drives, followed by a ninth extra-stimulus (S2) drive; the CL of the S1 train was fixed at 250 ms, and S2 stimuli gradually decreased from 100 ms at 10 ms or 2 ms step until no action potential duration after the S2 or atrial arrhythmias occurred. In arrhythmia induction, both PES and burst pacing (2 ms pulses at 50 Hz, 2 s burst duration, no more than six times) were used to determine susceptibility to atrial arrhythmia. AAs are defined as those that last for >2 s. To analyze the susceptibility to either pacing modality, we grouped induced AA durations into those lasting no more than 10 s, between 10 s and 30 s, and >30 s. We also grouped the morphology of AAs into monophasic or bipolar phase.

### Multielectrode Array Measurements

After MAP analysis, we used MEA technology to record the extracellular electrophysiological status and calculate excitation conduction velocity (*n* = 6 in each group). An array of 32 unipolar electrodes (each inter-electrode distance 500 μm) covered a piece of the LAA atrium square (3 × 3 mm). The map signals were recorded simultaneously and processed by a computer, and the parameters were acquired and analyzed using Cardio 2D + software (Multi Channel Systems, Reutlingen, Germany). The TAT was defined as the delay time between the first positive peak of field potential and the last activation electrode. The dispersion of TAT was evaluated by the standard deviation of TAT at 32 electrodes in LAA. The CV of the excitation transmission within the MEA registration area was also recorded.

### Immunohistochemistry

Under the anesthetization of pentobarbital sodium (40 mg/kg, Sigma), rats’ hearts (*n* = 5 in each group) were harvested for immunohistochemistry and Masson’s trichrome to assess collagen deposition. All four groups of myocardial histological specimens were fixed in 4% paraformaldehyde embedded in paraffin and sliced into sections of 4 micrometers from the paraffin block of the LA. The sections were stained with tumor necrosis-alpha (TNF-α, monoclonal rabbit anti-TNF-α antibody; 1:200), interleukin-6 (IL-6, monoclonal rabbit anti-IL-6 antibody; 1:200), and interleukin-1 (IL-1, monoclonal rabbit anti-IL-1 antibody; 1:200). The sections were then incubated with a secondary antibody (goat anti-mouse antibody; 1:50). We determined density by computer-assisted Image-Pro Plus software (Media Cybernetics, Rockville, MD, United States). Each slide was examined under a fluorescence microscope with a 200× objective to select three fields. Areas of inflammatory cytokines were colored red, and the computer automatically measured the positive areas. The ratio of the red areas to the total detected area was defined as the density. The mean density in the three selected fields was used to present the average density.

### Western Blot Analysis

Western blotting was conducted on lysates from frozen tissues (*n* = 3 in each group) as published to assess the relative protein levels of Cx40 and Cx43 (Shi et al., 2017b). According to our previously described methods, antibodies included Cx40 (1:1000; Abcam, Cambridge, United Kingdom), Cx43 (1:1000; Abcam), TNF-α (1:1000, Abcam), TGF-β (1:1000, Proteintech), p-Smad2 (1:1000; CST), Smad2 (1:2000, CST), p-Smad3 (1:1000; CST), Smad3 (1:2000, CST), MMP-9 (1:500, Abcam), TIMP-1 (1:1000, Proteintech), Collagen I (1:1000, Abcam), Collagen III (1:500, Abcam), and α-SMA (1:10000, Abcam). The expression of S1R (1:1000; Abcam) was measured in the atrium and hippocampus, and β-actin served as a housekeeping reference protein. Western blot film images were detected and calculated using ImageJ software (NIH, Bethesda, MD, United States).

### Fibrosis Quantification

The left atrial atriums the paraffin sections of four groups were fixed and stained with Masson’s trichrome (*n* = 5 in each group). The collagen depositions were dyed blue and analyzed by a quantitative digital image analysis system (Image Pro-Plus, version 6.0, Media Cybernetics, Inc.). The three fields of the LAA were averaged to assess the collagen fraction (*n* = 5 in each group).

### Statistical Analysis

Continuous variables and proportions were expressed as the mean ± standard error and percentage, respectively. Student’s *t*-test or Pearson’s chi-squared test were used to assess the differences of two groups. One-way ANOVA followed by Tukey’s multiple comparison test or two-way ANOVA followed by Sidak’s multiple comparison test were used to evaluate group differences. Statistical significance was defined as *P* < 0.05.

## Results

### Behavioral Measurements

Results from the forced swim test showed that MDS rats exhibited shortening of prolonged immobility times compared to the MDD group (Figure 1B). The sucrose preference results showed significantly reduced sucrose preference in the MDD rats, with MDS rats exhibiting a significant increase in sucrose preference compared with the MDD rats after the fourth week of CUMS (Figure 1C). Finally, body weights was significantly impacted by the group and the group-by-time interaction. At the end of the first week, four groups showed no significant difference, but MDD rats lost weight at the end of the following 3 weeks; the MDS group was relatively heavier than the MDD group in the same period (Figure 1D). Therefore, MDD significantly reduced sucrose preference and body weight while increasing immobility time, indicating the success of the depression model construction. SA4503 partially alleviated these depressed behaviors.

### SA4503 Alleviated Atrial Electrical Remodeling

**FIGURE 2:**
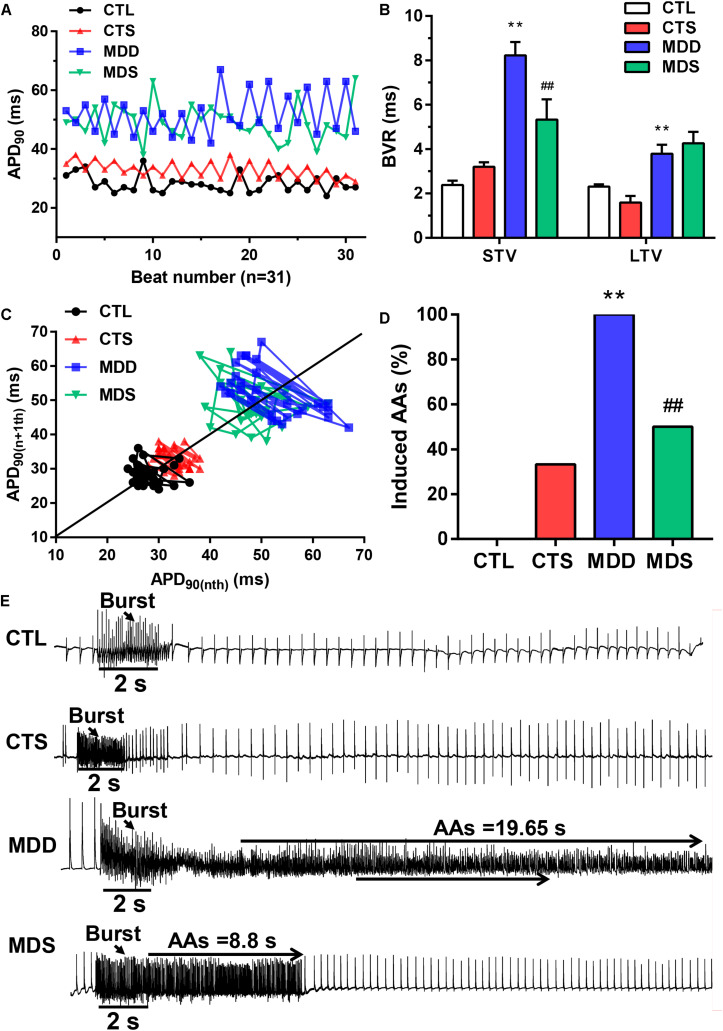
Representative dynamic changes in consecutive 31 APD90 and AA vulnerability. **(A)** Poincaré plots showed a representative increase in BVR in the MDD group. **(B)** The mean STV and LTV were larger in the MDD group than in the CTL group. **(C)** The *P* value for the MDD group vs. the CTL group. **(D)** AAs were induced in the MDD group, while AAs were not induced in the CTL group. Three out of six rats were induced in the MDS group. **(E)** Representative recording of burst pacing in the four groups (*n* = 6). ^∗∗^*P* < 0.01 vs. CTL group; ^##^*P* < 0.01 vs. MDD group.

During S1S1 dynamic pacing, BVR, STV, and LTV measured the heart rate and its stability. The STV in the MDD group was significantly higher than that in CTL group (STV: 8.23 ± 0.60 ms vs. 2.38 ± 0.20 ms, *P* < 0.01); LTV in MDD rats showed a similar result (LTV: 3.79 ± 0.41 ms vs. 2.31 ± 0.10 ms, *P* < 0.01, Figures 2A–C). The incidence of AAs was 100% in the MDD group but only 50% in the MDS group (*P* < 0.01, Figures 2D,E and Table 1) during burst stimulus. Monomorphic AAs with a long-lasting duration were more common in the MDD group during PES or burst stimulus compared to the MDS group, which indicated high sensitivity of induced AA. SA4503 alleviated atrial electrical indexes.

**TABLE 1:** Incidence of AAs induced by PES or burst pacing.

	**CTL (*n* = 6)**	**CTS (*n* = 6)**	**MDD (*n* = 6)**	**MDS (*n* = 6)**
**Inducibility**				
PES	33.33% (2/6)	16.67% (1/6)	66.67% (4/6)	33.33% (2/6)
Burst stimuli	0	33.33% (2/6)	100% (6/6)	50% (3/6)
No response	66.67% (4/6)	50% (3/6)	0	16.67% (1/6)
**Duration**				
<10 s	33.33% (2/6)	16.67% (1/6)	83.33% (5/6)	33.33% (2/6)
10–30 s	0	33.33% (2/6)	33.33% (2/6)	16.67% (1/6)
>30 s	0	0	16.67% (1/6)	33.33% (2/6)
**CL of AAs**				
Mean CL (ms)	53.19 ± 1.19	60.82 ± 15.78	49.17 ± 2.83	56.88 ± 11.80
Shortest CL (ms)	37 ± 4	48.33 ± 11.56	38.75 ± 3.70	50 ± 6.16
Longest CL (ms)	83.5 ± 1.5	67 ± 18.78	65.5 ± 12.34	75.4 ± 12.8
**Type of AAs**				
Mono	33.33% (2/6)	33.33% (2/6)	83.33% (5/6)	50% (3/6)
poly	0	16.67% (1/6)	33.33% (2/6)	33.33% (2/6)

*PES, programmed electrical stimulation; CL, cycle length; AAs, atrial arrhythmias.*

### SA4503 Restored Cardiac Conduction

**FIGURE 3:**
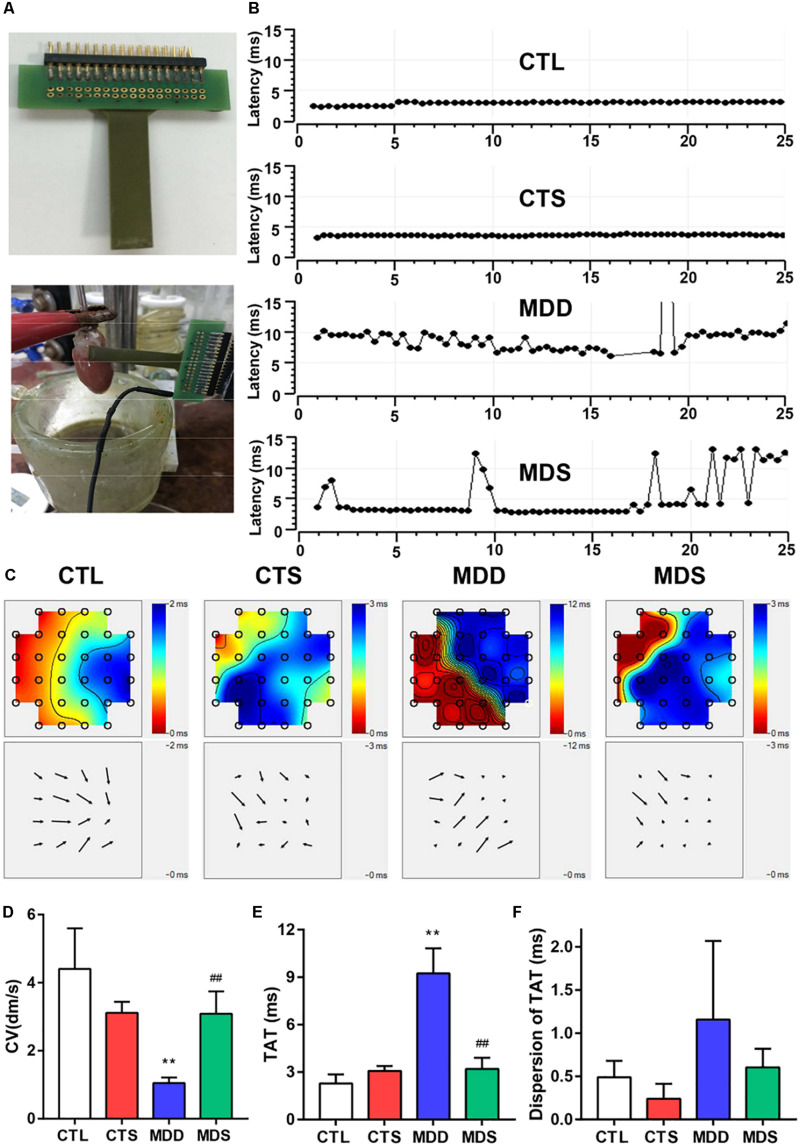
SA4503 improved the left atrial conduction function after CUMS. **(A)** Representation of an MEA electrode. **(B)** Representative examples of conduction heterogeneity map in the left atrium. Conduction was more heterogeneous in rats with MDD compared to the MDS rats. **(C)** Representative examples of the isochronous map in the left atrium by MEA recordings. Areas of isochronal crowding were found in rats with MDD. The degree of crowding decreased in the MDS rats (*n* = 6). **(D)** Changes in CV in each group. **(E)** Long-term trace of instantaneous beating TAT. **(F)** The dispersion of the dots represents the dispersion of TAT. CUMS, chronic unpredictable mild stress; MEA, multielectrode array; TAT, total activation time; CV, conduction velocity. ^∗∗^*P* < 0.01 vs. CTL group; ^##^*P* < 0.01 vs. MDD group.

Figure 3A shows the 32 monopolar electrodes and its covering of the LAA. Figure 3B shows the spontaneous real time LAA surface latency recordings in the four groups. LAA activation maps demonstrated the conduction from the fastest area (dark red) to the slowest area (dark blue), pointed out by isochrones and arrows (Figure 3C). In addition, CV, TAT, and the dispersion of TAT reflected homogeneity of conduction (Figure 3D). In the CTL group, the excitation spread quickly in the MEA registration, and the TAT was slow (2.28 ± 0.23 ms) (Figure 3E). After CUMS, the MDD group showed a significant decrease compared to the co-administration of SA4503 (1.05 ± 0.06 dm/s vs. 3.09 ± 0.26 dm/s, *P* < 0.01) while the TAT was increased (9.23 ± 0.63 ms vs. 3.19 ± 0.29 ms, *P* < 0.01). Moreover, the dispersion of TAT showed no significance whether in the CTL group vs. MDD group or in the MDD group vs. MDS group (Figure 3F).

### SA4503 Partially Improved Myocardial Inflammation

**FIGURE 4:**
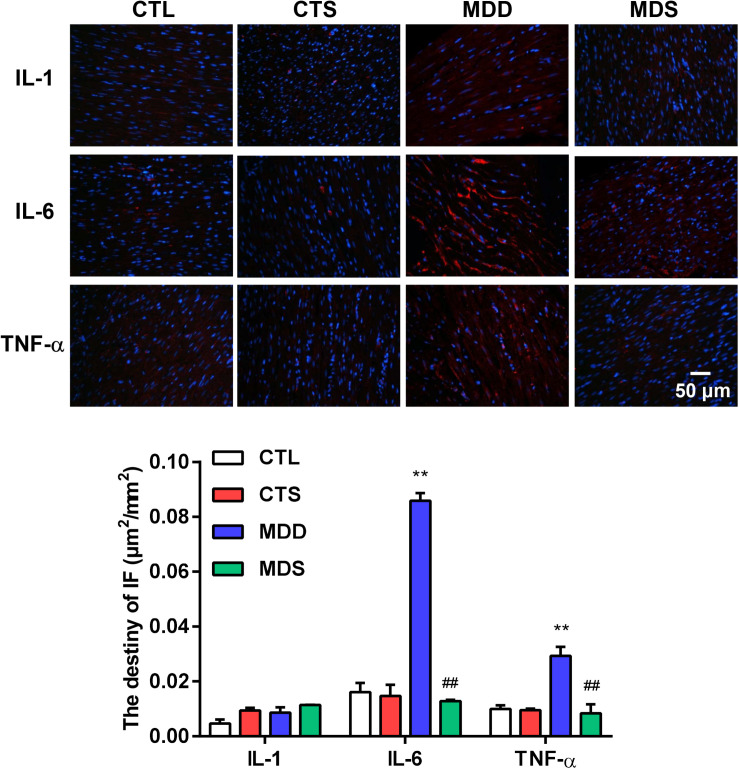
Histological results for IL-1, IL-6, and TNF-α in four groups. Representative images of IL-1, IL-6, and TNF-α immunofluorescence staining (original magnification, 400 ×) of LA (*n* = 5). Blue: Nucleus of cardiomyocyte; Red: IL-6 positive, IL-6 positive, TNF-α positive. The lower panel shows the statistical bars of IL-1, IL-6, and TNF-α MDD group. LA = left atrium. ^∗∗^*P* < 0.01 vs. CTL group; ^##^*P* < 0.01 vs. MDD group.

**FIGURE 5:**
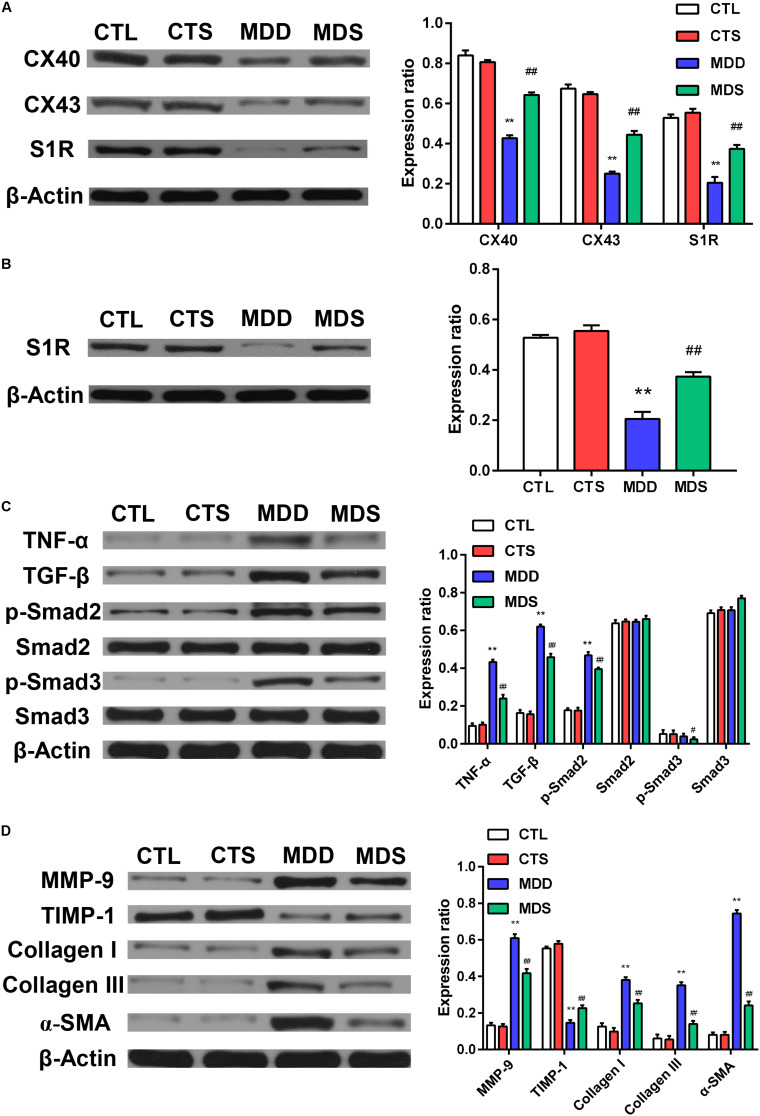
Quantification of the expression levels of gap junction and inflammatory pathways, collagen disposition, and S1R proteins. **(A)** The MDD group showed downregulation of Cx40, Cx43, and S1R in left atria. The MDS group showed partial reversals of downregulation (*n* = 3). **(B)** The MDD group showed downregulation of S1R in the hippocampus and the MDS group showed partial reversals of these regulatory changes (*n* = 3). **(C)** The MDD group showed upregulation of TGF-β/Smad2/3 pathway. **(D)** The MDD group showed more collagen disposition. S1R = sigma-1 receptor. ^∗∗^*P* < 0.01 vs. CTL group; ^#^*P* < 0.05, ^##^*P* < 0.01 vs. MDD group.

Figure 4 represents the changes in the LAA levels of IL-1, IL-6, and TNF-α. Compared to the CTL group, the MDD group displayed a significant increase in IL-6 and TNF-α (*P* < 0.01 for Figure 4); the levels of IL-6 and TNF-α in the MDS group were significantly decreased after administering SA4503 during CUMS (*P* < 0.01), while the level of IL-1 was not statistically significant between the four groups. The expression of TNF-α was mainly medicated by the TGF-β as a core molecule to the downstream Smad2/3 signaling. SA4503 significantly decreased the high level of TGF-β/Smad2/3 signaling expression as well as MMP expression, which was the genesis of atrial fibrosis (Figure 5C).

### SA4503 Decreased Fibrosis and the Increased Expression of Gap Junction Proteins in the Atrium

**FIGURE 6:**
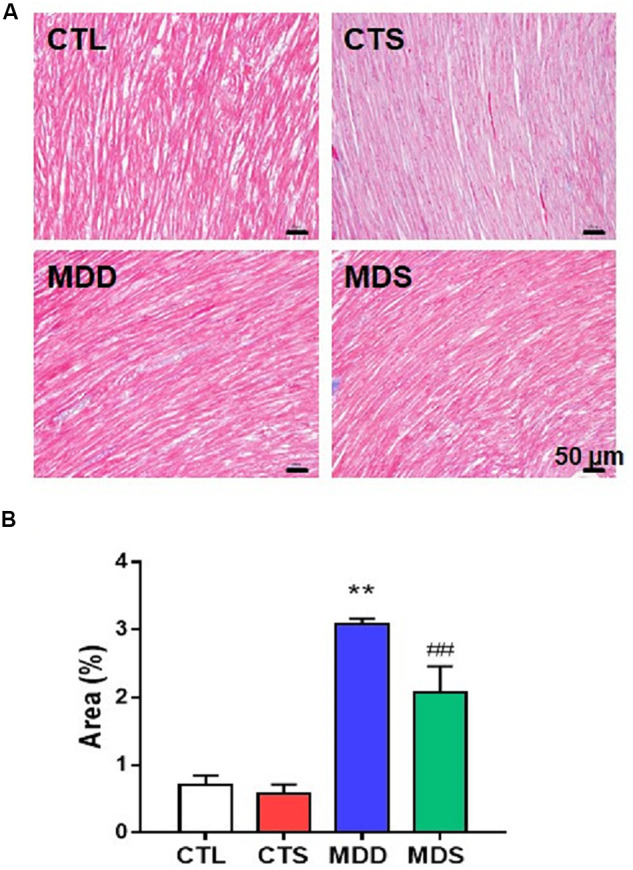
Atrial myocardial fibrosis. **(A)** Atrium slices stained with Masson stain (original magnification: 200×). **(B)** Quantification of the fibrotic area in each group (*n* = 5). ^∗∗^*P* < 0.01 vs. CTL group; ^##^*P* < 0.01 vs. MDD group.

Figures 5A,D show the western blot analysis of gap junctions, including Cx43 and Cx40, collagen disposition, and S1R proteins. Results revealed that Cx40, CX43, and S1R protein levels decreased in the MDD rats compared to the control rats (all at *P* < 0.01). The S1R expression in hippocampus showed the similar results Figure 5B. Meanwhile, SA4503 suppressed the downregulation of the three proteins that were induced by CUMS. Also, MMP-9, TIMP-1, Collagen I, Collagen III, and α-SMA protein levels increased in the MDD group compared to the MDS group and the CTL group (all at *P* < 0.01). The MDD group showed higher collagen disposition than that in the CTL group (Figure 6).

## Discussion

**FIGURE 7:**
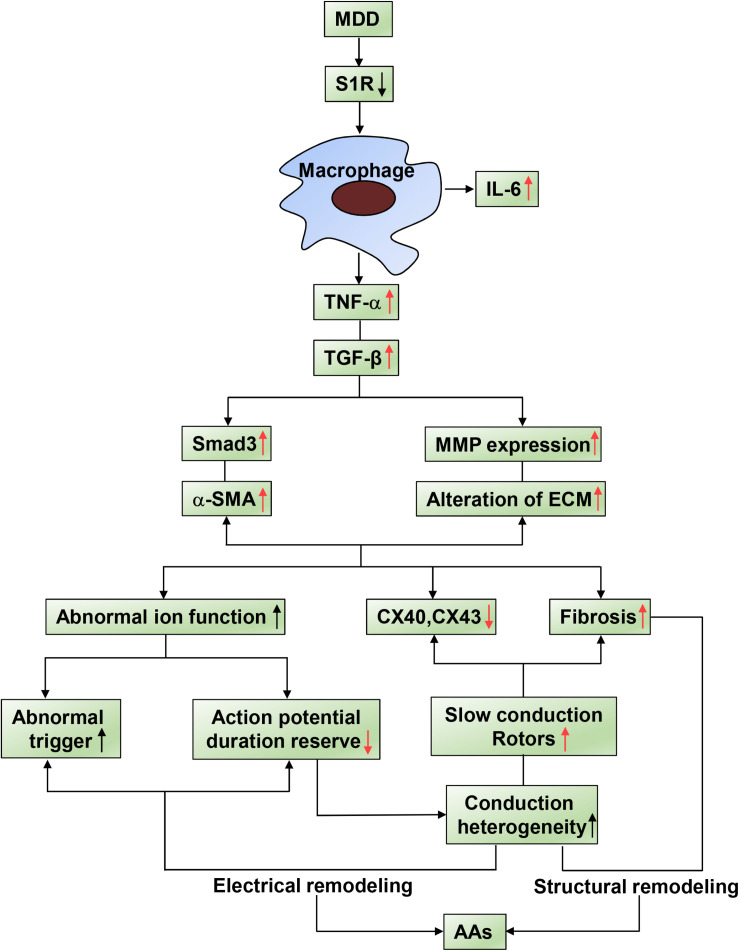
Summary of schematic showing of the reversal effect of SA4503 effects on AA. Inflammatory factors causing AA substrate. Activated immune cell relates AA to TNF-α and TGF-β/Smad2/3 pathway causes altered ion functions, downregulation of Cx40 and Cx43, and atrial fibrosis.

Based on our previous findings, which are indicated by black arrows in Figure 7, activation of S1R alleviated atrial autonomic nerve function and AF incidence after 4-week CUMS. However, to reveal a more complete mechanism in our systemic research, we further investigated the relationship between inflammations and conduction junctions after co-administration of SA4503, shown by red arrows in Figure 7. In this study, the Sigma-1 receptor (S1R) agonist, SA4503, improves atrial myocyte inflammation and conduction junctions of depression disorder via TGF-β/Smad2/3 signaling. By decreasing the TGF-β downstream cascade, myocardial inflammation, atrial conduction velocity, excitation propagation disorder, and myocardial inflammation are significantly recovered. SA4503 may be the promising pharmacological agent to treat depression related AA (Figure 7).

### SA4503 and Electrical Remodeling

A reduced repolarization reserve could be identified by several indicators including BVR, STV and LTV (Thomsen et al., 2006; Hinterseer et al., 2009). Without the requirement of heart rate stability, BVR distributed on a high baseline, and prolonged STV as well as LTV are highly associated with a reduced repolarization reserve (Flore et al., 2011). Consistent with our previous study, the repolarization of a single cell may affect the behavior of multiple ion currents, resulting in BVR turbulence. It is widely acknowledged that slight changes in ion fluxes in the plateau phase significantly affect the membrane potential and thus the duration of AP (Liu X. et al., 2018). PES or burst stimulation are performed to induce reentry, which may be the one of the main causes of atrial arrhythmias. Burst pacing and single extra stimulus were conducted to provoke AAs, inducing reentry as the primary electrophysiologic mechanism of arrhythmias (Matsuyama et al., 2013). After continuous intraperitoneal administration of SA4503 for 28 days, the MDS group shows a decreased incidence of AAs and short duration of BVR compared to the MDD group, indicating its anti-arrhythmia effects and stability of repolarization. The instability of repolarization displayed by the above reported high values of BVR may also be a pathogenesis of AAs. The repolarization heterogeneity in the LAA lead to cardiac conduction dysfunction and even reentry excitation (Verrier et al., 2016).

### SA4503 and Cardiac Conduction

Our findings illustrate that the MDD group exhibited heterogeneous spread of excitation and the equipotential lines of velocity are non-uniform and disorderly. Excitement can be partially transmitted from one region of atrial tissue area to another in a no-uniform direction opposite to its main direction of propagation. The initial point of excitement is formed like tornados, called rotors, and the process of transmission is known as reentry. Although controversy exists regarding the mechanisms for the initiation of AA episodes, increasing evidence supports reentry as the dominant mechanism maintaining arrhythmia after onset (Amit et al., 2010). Wavelength theory suggests that reentry can be disrupted by increasing either the CV or the tissue refractory properties (Igarashi et al., 2012). Electrical and structural remodeling severely interfere with impulse transmission (Makowiec et al., 2018), contributing to abnormal electrical signal propagation. The lack of electrical connection contributes to desynchronized cardiac activation and contraction, as in connexins 40 and 43, the principle atrial gap junction proteins (Davis et al., 1994). Consequently, SA4503 may significantly improve myocardial CV to eliminate reentry, which requires either increasing tissue excitability or increasing levels of intercellular connectivity, controlled predominately by gap junctions.

### SA4503 and Myocardial Inflammation

TGF-α and IL-6 are localized in atria more frequently in AAs (Yamashita et al., 2010). Atrial fibrosis promotes atrial arrhythmogenesis via multiple mechanisms: fibrosis and collagen deposition impair cell-to-cell coupling, causing homogeneities and asymmetries in conduction, creating the potential for unidirectional block and reentrant proarrhythmia. Serum levels of interleukin-6 (IL-6) were independently associated with AA risk, becoming an effective inflammatory biomarker for future predictions (Marcus et al., 2008). TNF-α is secreted mainly by activated macrophages and is considered an inflammatory biomarker in the development of AA by pathologically promoting atrial fibrosis, altering the expression or distribution of gap junction proteins, affecting intracellular calcium homeostasis, and triggering abnormal electrical activity that leads to atrial dilatation and conduction heterogeneity (Hu et al., 2015). IL-1 was also found to play a direct role in AAs by mediating proinflammatory electrical remodeling (Liu Y. et al., 2018). In this study, we found that the activation of S1R decreases the elevated levels of TNF-α and IL-6 in MDD rats. Similarly, results from a recent article have reported that peak serum TNF-α and IL-6 were significantly increased in S1R knock out mice (Rosen et al., 2019). In addition, the increased level of TNF-α induced the secretion of TGF-β, regulating the downstream Smad3 pathway, and causing the high expression of α-SMA. TGF-β induced fibroblasts to differentiate into myofibroblasts, express α-SMA, and participate in the formation of ECM. We speculate that the effect of this on the levels of inflammatory cytokines are attributable to the 4-week administration of SA4503.

In this study, we did not conduct some experiments on invading immune active cells, but we expect to further investigate this in the near future. Based on previous research, immune cells including lymphomononuclear cells, macrophages, neutrophils, and mast cells infiltrate the atrial myocardium of AF patients. The acute and chronic inflammation might mediate cascades and signals, which aggravate the progress of AF (Hu et al., 2015). Taking a macrophage as an example, the LPS-stimulated macrophage increased the incidence of AF through inflammatory secretion as well as the atrial myocytes’ electrical remodeling by using co-culture with a LPS-stimulated macrophage (Tuomi et al., 2011). In summary, inflammatory cells play vital roles in the atrial remodeling, occurrence, and development of AF after depression, and all these hypotheses need to be proven.

### SA4503 and the Expression of Gap Junction Proteins

In this study, we report that Cx40 and Cx43 expression levels are significantly reduced in depression model rats, which could be reversed by SA4503. Numerous studies have found that rats exposed to CUMS exhibited depression-like behavior as well as a reduction in Cx43 expression and Cx43 puncta density in the prelimbic cortex. This phenomenon is consistent with the association between gap junction and extracellular matrix deposition of collagen in depression. Myocardial conduction rate is partially determined by the gap junction protein distribution and ECM changes. Cx43 mutant mice are more prone to develop spontaneous AA (Tuomi et al., 2011). Clinical studies showed that Cx43 expression in patients with AA was dramatically reduced, suggesting that Cx43 may be one of the important mechanisms in pathogenesis of AA (Kostin et al., 2002). Tomonori Igarashi et al. found decreased expression of Cx43 in pig model of atrial fibrillation. After 7 days of specific Cx43 gene therapy, the pigs’ atrial conduction returned to a normal standard (Igarashi et al., 2012). It is known that MMPs are involved in atrial remodeling and TGF-β alter the ECM gene expression of MMP. In this study, we found that, in depressed rats, MMP protein levels increased while Cx43 and Cx40 protein expression levels decreased and atrial conduction slowed; SA4503 treatment reduced the expression of MMPs and preserved the level of Cx43 and Cx40.

## Clinical Implications

The S1R is involved in the control of gap junction proteins, inflammatory factors, and NMDA receptor (receptors of N-methyl-D-aspartic acid) stimulation related to remodeling (Skrzycki and Czeczot, 2014). The study of mechanisms of SA4503 on atrial arrhythmia is fully rounded. The study demonstrates that SA4503 improves myocardial inflammation and conduction velocity by TGF-β/Smad2/3 signaling in depressed rats. These results support a link between the S1R and anti-inflammation with the downstream anti-arrhythmia. Currently, SA4503 is only used for experimental studies as a Sigma-1 receptor agonist and not for clinical patients. We checked relevant instruction and published literature, but unfortunately there is little direct evidence of side effects. Fluvoxamine is the potent agonist of the Sigma-1 receptor as well as a serotonin uptake inhibitor, which is widely used in the clinic. We speculate that SA4503 might possible have similar side effect medication due to the semblable structure (Bhuiyan et al., 2010). A meta-analysis reported that fluvoxamine had fewer side-effects compared to tricyclics, including hypotension, bradycardia, sweating, nausea, weight loss, constipation, and agitation (Omori et al., 2009). All in all, SA4503 has promising clinical applications, but its safety needs to be carefully demonstrated.

## Study Limitations

A disputed question remains regarding the relationship between the expression of gap junctions, and AF. Polontchouk et al. found that, in human atria there was no significant difference in Cx43 expression between the AA group and the control group, however, a 2.7-fold increase in the expression of Cx40 was found in AAs, which was similar to that in isolated rat atria (Polontchouk et al., 2001). The abnormal expression and distribution of atrial Cx40 and Cx43 were also observed with alterations in atrial CV. This abnormal trigger and conduction heterogeneity may lead to non-uniform electrical coupling, contributing to transmission dispersion and atrial arrhythmia. In this case, we need to investigate the distribution of Cx43 and Cx40 proteins to clarify the clinical significance of gap junction remodeling in AA after depression.

## Conclusion

The present study shows that SA4503 ameliorates excitation propagation disorder, increases atrial conduction velocity, and improves inflammatory factors by TGF-/Smad2/3 signaling in a rat depression model. Myocardial inflammation, atrial electrical remodeling, conduction heterogeneity, and low expression levels of gap junction proteins are identified as potential mechanistic contributors.

## Data Availability Statement

The datasets generated for this study are available on request to the corresponding authors.

## Ethics Statement

The animal study was reviewed and approved by All experimental procedures were conducted in accordance with the Guide for the Care and Use of Laboratory Animals published by the United States National Institutes of Health (NIH Publication No. 82-23, revised 1996; Bethesda, MD, United States) and were approved by the animal experimental administration of Wuhan University, China (No. 20160103).

## Author Contributions

The present study was performed by all authors. XL, DH, and BY designed the study. XL, CQ, TY, LW, SL, CZ, and JL carried out the experiments. XL, CQ, SS, and JL analyzed the data. XL and DH wrote the manuscript. BY, CQ, SS, and CZ revised the manuscript. All authors were responsible for the final content.

## Conflict of Interest

The authors declare that the research was conducted in the absence of any commercial or financial relationships that could be construed as a potential conflict of interest.
