# Corrigendum: The Reversal Effect of Sigma-1 Receptor (S1R) Agonist, SA4503, on Atrial Fibrillation After Depression and Its Underlying Mechanism

**DOI:** 10.3389/fphys.2022.846676

**Published:** 2022-04-05

**Authors:** Xin Liu, Chuan Qu, Shaobo Shi, Tianxin Ye, Linglin Wang, Steven Liu, Cui Zhang, Jinjun Liang, Dan Hu, Bo Yang

**Affiliations:** ^1^ Department of Cardiology, Renmin Hospital of Wuhan University, Wuhan, China; ^2^ Cardiovascular Research Institute, Wuhan University, Wuhan, China; ^3^ Hubei Key Laboratory of Cardiology, Wuhan, China

**Keywords:** sigma-1 receptor, depression, conduction, inflammation, atrial arrhythmia

In the original article, there was a mistake in “Figure 6” as published. The figure was mis-uploaded, and different from the versions we submitted originally. The corrected “Figure 6” appears below.

**FIGURE 6:**
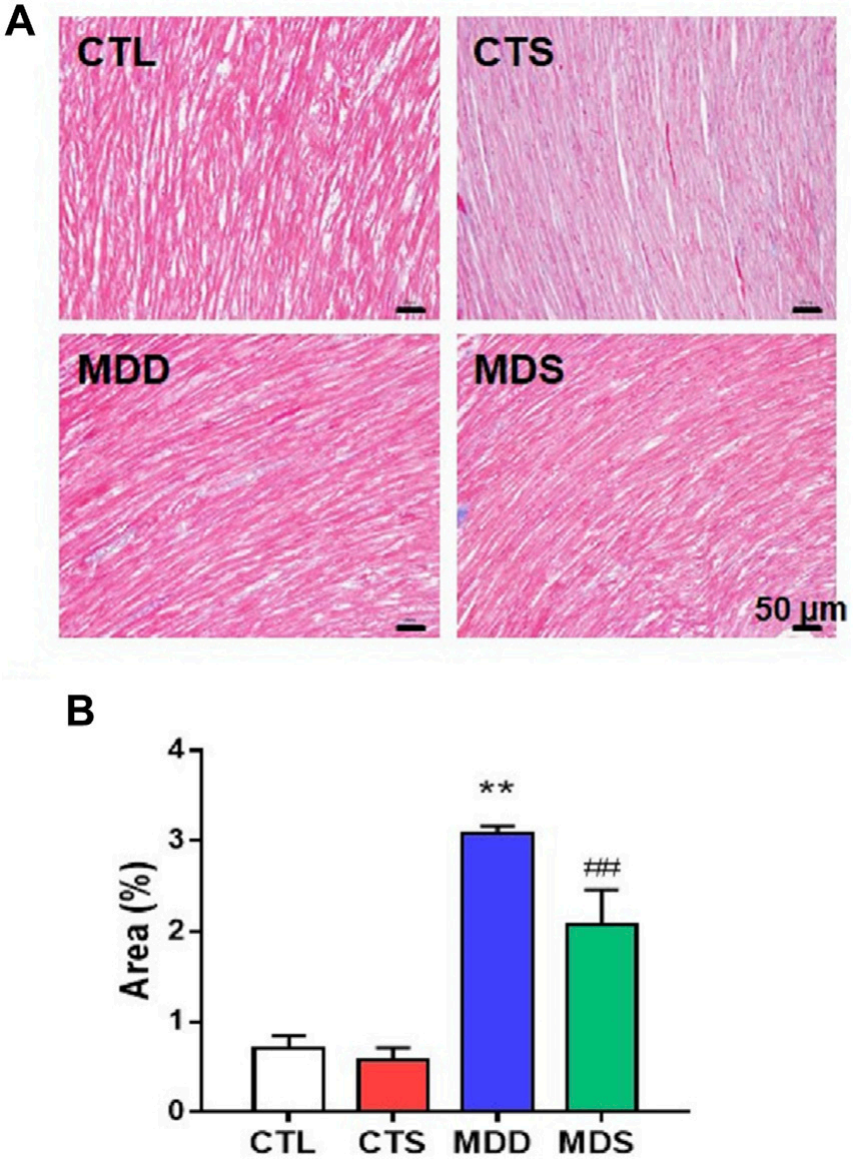
Atrial myocardial fibrosis. **(A)** Atrium slices stained with Masson stain (original magnification: ×200). **(B)** Quantification of the fibrotic area in each group (*n* = 5). **p < 0.01 vs. CTL group; ##*p* < 0.01 vs. MDD group.

The authors apologize for this error and state that this does not change the scientific conclusions of the article in any way. The original article has been updated.

